# Why Small Is Beautiful: Wing Colour Is Free from Thermoregulatory Constraint in the Small Lycaenid Butterfly, *Polyommatus icarus*

**DOI:** 10.1371/journal.pone.0122623

**Published:** 2015-04-29

**Authors:** Rien De Keyser, Casper J. Breuker, Rosemary S. Hails, Roger L. H. Dennis, Tim G. Shreeve

**Affiliations:** 1 Centre for Ecology, Environment and Conservation, Department of Biological and Medical Sciences, Faculty of Health and Life Sciences, Oxford Brookes University, Oxford, OX3 0BP, United Kingdom; 2 Evolutionary Developmental Biology Research Group, Department of Biological and Medical Sciences, Faculty of Health and Life Sciences, Oxford Brookes University, Oxford, OX3 0BP, United Kingdom; 3 Centre for Ecology and Hydrology, Maclean Building, Wallingford, OX10 8BB, United Kingdom; University of Kent, UNITED KINGDOM

## Abstract

We examined the roles of wing melanisation, weight, and basking posture in thermoregulation in *Polyommatus Icarus*, a phenotypically variable and protandrous member of the diverse Polyommatinae (Lycaenidae). Under controlled experimental conditions, approximating to marginal environmental conditions for activity in the field (= infrequent flight, long duration basking periods), warming rates are maximised with fully open wings and maximum body temperatures are dependent on weight. Variation in wing melanisation within and between sexes has no effect on warming rates; males and females which differ in melanisation had similar warming rates. Posture also affected cooling rates, consistent with cooling being dependent on convective heat loss. We hypothesise that for this small sized butterfly, melanisation has little or no effect on thermoregulation. This may be a factor contributing to the diversity of wing colours in the Polyommatinae. Because of the importance of size for thermoregulation in this small butterfly, requirements for attaining a suitable size to confer thermal stability in adults may also be a factor influencing larval feeding rates, development time and patterns of voltinism. Our findings indicate that commonly accepted views of the importance of melanisation, posture and size to thermoregulation, developed using medium and large sized butterflies, are not necessarily applicable to small sized butterflies.

## Introduction

The colour and pattern of butterfly wings has been described as being shaped by three major selective forces: thermoregulation, apparency to conspecifics and reduction of predation risk [1–11]. These requirements can conflict [12] as apparency may promote more brightly coloured butterflies [2], [10], [13]-[14], but these individuals may be more visible to predators [9], [14–17] and generally heat up more slowly, reducing the time they can be active [1], [18–21]. Some butterflies have overcome some of these constraints by uncoupling pattern formation on the dorsal and ventral wing surfaces, reducing conflicts between apparency and predation risk [9], [22]. For butterflies that adopt a closed-wing basking posture (lateral baskers), the dorsal surfaces may be primarily devoted to apparency and the ventral to defence and thermoregulation, but for open wing baskers the dorsal surface may be involved in apparency, thermoregulation and defence, and the ventral surface primarily involved in defence [12], [21]. Heating and cooling rates are partly dependent on microclimate and the interaction of this with wing colouration, basking posture, basking method and body size [1], [3], [23]-[24]; wing melanisation has been demonstrated to have a significant role in thermoregulation by medium and large sized butterflies (>c. 60 mg and >c. 45 mm wingspan).

Small sized bodies have a low capacity for heat retention, raising the intriguing question of how much of a contribution wing melanisation has in their thermoregulation and whether their thermal stability is below a threshold that wing melanisation can affect. If this is the case, wing colour and pattern variation will be relatively free from thermal constraints.

The Lycaenidae are the most species-rich group of butterflies [7] and within this group, the polyommatine section [25] is characterised by an extremely high degree of variability in morphological traits [26] with a comparatively high number of species. The colour and pattern on the wings of lycaenid butterflies play an important role in mate recognition [7] and small scale reflective differences are used for mate recognition [11].

The common blue butterfly, *Polyommatus icarus*, is a small sized (c. 30mg and c.35 mm wingspan), dorsal basking member of the polyommatine section. Previous studies on thermoregulation in *P*. *icarus* [27] indicate that thoracic temperatures at take-off differ between geographic locations, and within these locations take-off temperatures are similar for males and females which differ in wing colour. Additionally, body temperatures of this species in the field are more closely related to basking site temperature than to solar radiation intensity [27]-[28]. The latter would be expected to be a key factor if the wings play a role in thermoregulation. Therefore we hypothesise that body size is a more important factor in thermoregulation than wing colour and explore these relationships using controlled laboratory experiments. Our results are consistent with this hypothesis and we put our findings into an ecological context.

## Materials and Methods

### Heating and cooling rates

**Fig 1:**
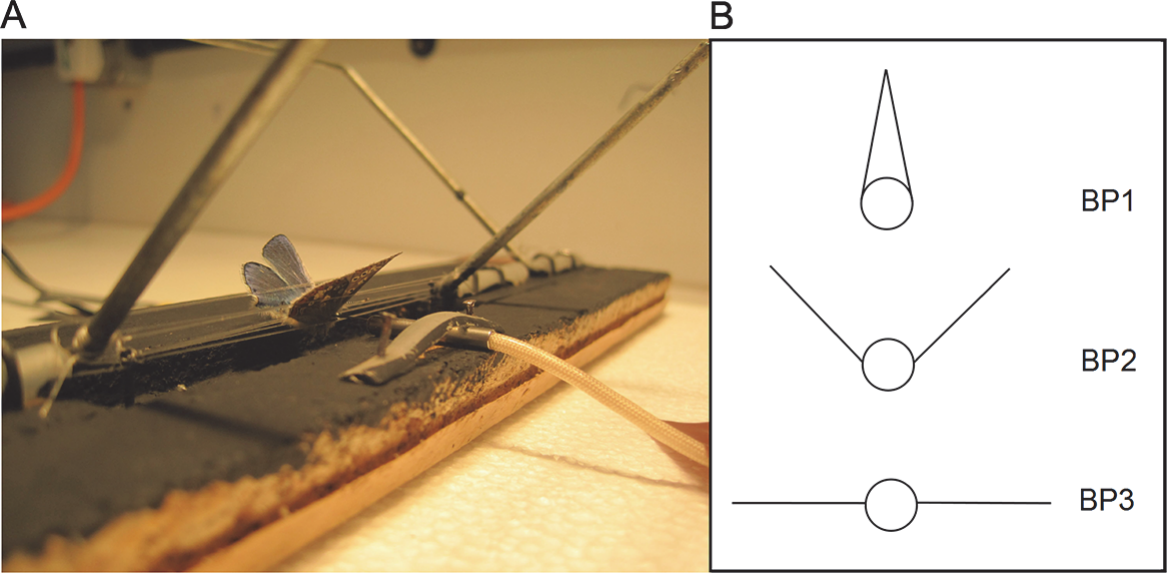
Frame with *Polyommatus icarus* male with partially opened wings (basking position 2) used to determine thoracic temperatures during heating and cooling experiments. The thermoprobe (front) is inserted in the thorax and held in place by a strap (a). Schematic representation of the three basking positions (b).

All experiments were conducted with *P*. *icarus* from laboratory stocks, originating from Frog Firle, Sussex (N: 50°47'33", E: 0° 8'23"), reared at 20°C, L16D8. In one set of experiments, freshly thawed individuals (21 females, 23 males) were used. These butterflies were previously killed by freezing immediately after wing drying on eclosion. In the second set of experiments, a smaller sample of live butterflies (12 females, 5 males) was used, within 24 h of eclosion, to investigate any differences in thermal properties between live and dead butterflies. All individuals were weighed to the nearest 0.001 g prior to testing (HR-120-EC, A&D Instruments). Heating and cooling rates were measured by inserting specimens in a frame (at 20°C in low light levels) with the butterfly body held in place with nylon lines (Fig 1A) allowing the wings (supported by the nylon lines) to be angled into different basking positions (Fig 1B). The frame with the butterfly was then placed under two 500 Watt halogen lamps (NR10461, Philips Lighting) positioned 0.8 m above the frame in a constant temperature room at 20 (± 1)°C. The average radiation load was 405.2 (± 6.7) Wm^-^², measured at body height, c. 30% greater than minimum radiation load at which *P*. *icarus* is active in the field [27]. The lamps had a higher (c. 30%) output in the near IR (700–900nm) than sunlight. The temperature next to the body (i.e. above the board surface) was 23.9 (± 0.8)°C, measured with a thermocouple shielded from the light source. Thoracic temperatures were recorded using a thermocouple (Type K, 0.04 mm diameter) inserted into the thorax, attached to a Physitemp BAT-12 digital thermometer. Temperatures of dead specimens were recorded every 5 seconds for a period of 5 minutes (S1 Table.). Most basking durations in the field for this butterfly are between 1 and 300 s [27]. To minimise the effect of transferring the framed butterfly from 20°C (room temperature) to 23.9°C (area under the lamp) and thus recording any rise in body temperature associated with moving the specimen to a warmer location, the first temperature reading was excluded from any analysis. After measurements of warming, the frame with the butterfly was shaded (39.8 ± 4.3 Wm^-2^) and thoracic temperatures were again recorded every 5 seconds for 5 minutes, with the first reading excluded from the analysis. For every dead individual, thoracic temperatures were measured for the three different wing positions (Fig 1B) in random order in one sequence to avoid damage to the specimen by repeated handling. Live butterflies were only tested for warming rates in two basking positions (BP1 and BP3) and body temperature was monitored for only two minutes, as most of the heating process happens in this period, identified from studies of dead butterflies. Additionally, because of voluntary movements in the frame they tended to change the area of wing exposed over time so tending to move fore- and hindwings together and thus exposing smaller wing areas to the lamp than at the start of each experimental run. Thus they could not be used for cooling rate measurements. Ambient temperature and light intensity were continuously measured with a data logger (Datahog2: Skye Instruments Ltd.). After testing, the wings of the dead butterflies were removed, illuminated with a fibre optic ring light (90 Watt) and photographed against a standard black and white background using a Nikon D1, set at ISO 200, F8, exp.1/320s, at a fixed focal length of 58 cm in a darkroom. Wings of the live experimental butterflies were not imaged because of scale losses due to their voluntary movements in the frame.

### Melanisation measurement

Wing darkness was determined using ImageJ [29]. Darkness equates to melanisation because the wing scales of Lycaenidae comprise of melanized basal scales [30] and non-melanized cover scales [31]-[32], with variation in the brightness of the wings of *P*. *icarus* being dependent on the ratio of these two scale types [27]. Melanisation (darkness) was measured in an area of 100 pixels on the basal part of both upper forewings following the method of [33], as the basal wing area is the most important in thermoregulation [34]. The brightness of the whole wing was estimated using a 200 pixel wing area centred on the middle of the wing. To standardise the overall colour of images before processing, colour (RGB) was first rescaled in Adobe Photoshop (CS5) adjusting the colour intensity within the “match colour” command (which does not affect brightness), to match each wing image against a standard black background. To standardise brightness values between samples, a melanisation index was calculated as:

$$1-\;\left(\left(G_{W}-B\right)/\left(W-B\right)\right)$$

where G_w_ = wing grey value measurement, B = black background grey value, W = white background grey values. This converted absolute values to a proportionate value of the total brightness scale (0 (= black) – 255 (= white)), reducing any effects of small changes in lighting conditions on measurements of wing melanisation. The average index values of left and right wings were then taken as a melanisation value for the individual. To estimate measurement error the left and right wings of 31 individuals were photographed twice and each photograph digitised twice (resulting in 8 measurements per individual). The analysis of imaging and digitising errors follows the method of [35].

### Analyses

Heating and cooling rates of every individual were taken from the slope of the regression between log time and thoracic temperature following the methods of [36], with unique rates being calculated for every wing position. For all individuals tested analysis (heating rates and cooling rates) was restricted to the first 2 minutes of the data series because after this period measured thoracic temperatures stabilised. The rates measured fitted the above regression model well (r^2^ >0.9, P<0.001 in all cases). Maximum body temperature, heating and cooling rates were analysed using general linear models. The effect of state (dead *vs*. live) was compared over the initial two-minute warming period using state, sex and basking position as explanatory variables. The effect of sex was determined using a model including sex (fixed effect) and individual nested within sex (random effect), the latter being the error term. The effect of basking position was determined by a model with sex (fixed effect), individual nested within sex (random effect) and basking position (fixed effect) using the interaction between basking position and individual as the error term (Model 1). The influence of weight was investigated by fitting a model with sex (fixed effect), basking position (fixed effect) and weight (covariate) (Model 2). Model 3 had sex and basking position as fixed effects and melanisation as a covariate. State (dead *vs*. alive) is not included in the three models as only dead individuals were used in these analyses. Quantitative comparison of the models (F ratio tests) was used to determine which components explained heating and cooling rates the most. Data were checked for normality using the Shapiro-Wilk’s normality test in R 2.15.3 [37].

### Ethics statement

*Polyommatus icarus* has no conservation designation in the UK. Individuals used to establish the breeding stock were taken from land owned by the National Trust with their permission.

## Results

**Fig 2:**
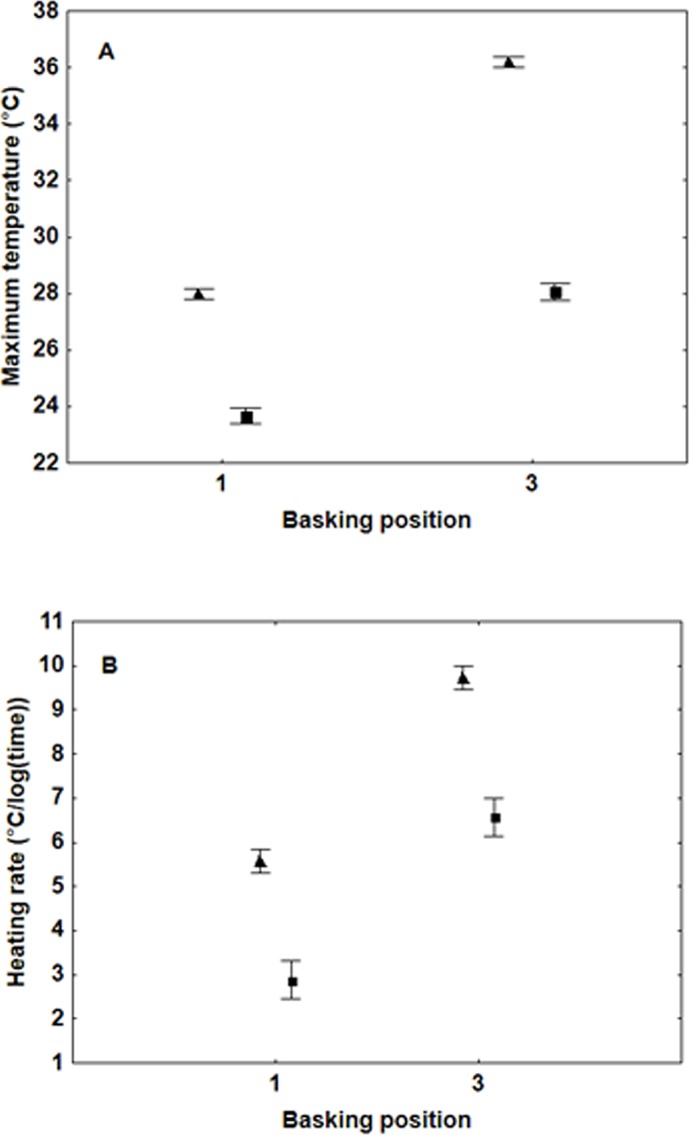
Maximum temperatures (basking position*state: F_1,64_ = 85.11, P < 0.0001) (A) and heating rates (basking position*state: F_1,64_ = 25.71, P < 0.0001) (B) for dead (▲) and live (▀) butterflies with wings closed (1) and open (3) (means +/- S.E.).

Dead individuals reached a higher maximum temperature and had faster heating rates than live ones, with the difference increasing the more open the wings (maximum temperature: basking position*state: *F*_1,64_ = 85.11, *P*<0.0001; heating rates: basking position*state: *F*_1,64_ = 25.71, *P*<0.0001; Fig 2). Despite these interactions, the pattern for heating rates between the basking positions for both live and dead butterflies is similar. There were no significant interactions between weight, sex and state in either comparison.

Measurement error of melanisation was negligible (Table 1). The melanisation index of males ranged from 0.48 to 0.62 and of females (darker) from 0.71 to 0.90. These are comparable with the values found for field collected individuals from the same populations (males: *t*_44_ = 1.449, *P* = 0.152; females: *t*_40_ = 0.783, *P* = 0.436). For both males and females there was a significant correlation between the darkness of the basal area and the main wing area (males: *r* = 0.486, *P* = 0.014; females: *r* = 0.697, *P* = 0.0005).

**Table 1:** Error analysis of melanisation measurements from 31 individuals of *P*. *icarus*, each imaged twice and each image digitised twice.

Source	SS	MS (x10^3^)	df	F	p
Individual	3.45963	115.32	30	22.18	<0.0001
Imaging error	0.16117	5.2	31	1.42	0.082
Residual = Digitising error	0.6812	3.66	186		

Imaging error is the error of photographing the wings and residual error is the error due to digitising. Model applied: melanisation = ind + imaging(ind).

**Fig 3:**
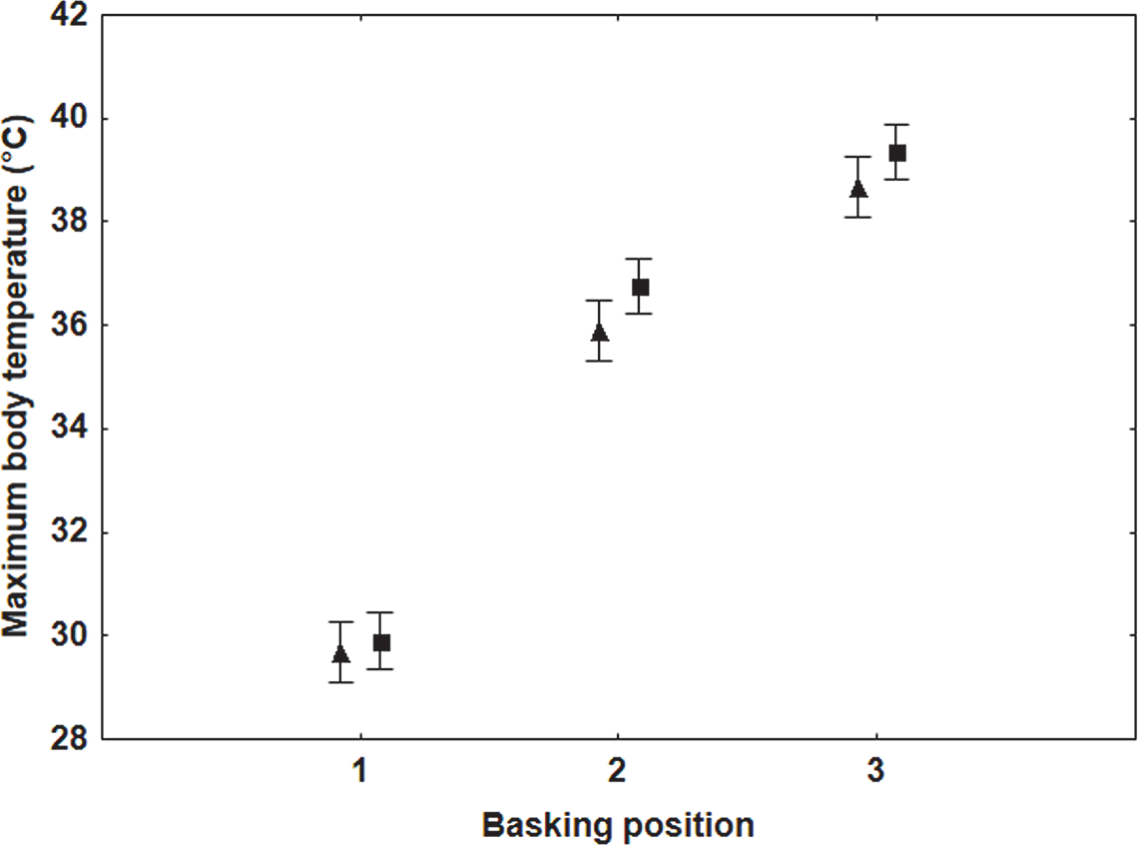
Maximum body temperatures (means +/- S.E.) for the three different basking positions (see Fig 1) for male (▀) and female (▲) *P*. *icarus* (basking position: F_2,86_ = 998.8, P < 0.0001).

Males reached a higher body temperature in all three basking positions than females (*F*_1,42_ = 13.1, *P* = 0.04). There was a highly significant effect of basking position on the maximum body temperature reached (*F*_2,86_ = 998.8, *P*<0.0001) the greatest temperatures being reached with the most open wings (Fig 3). Males and females did not differ in weight (*F*_1,42_ = 0.374, *P* = 0.544), but males had larger wings (*F*_1,42_ = 8.296, *P* = 0.006) and therefore slightly smaller bodies.

**Fig 4:**
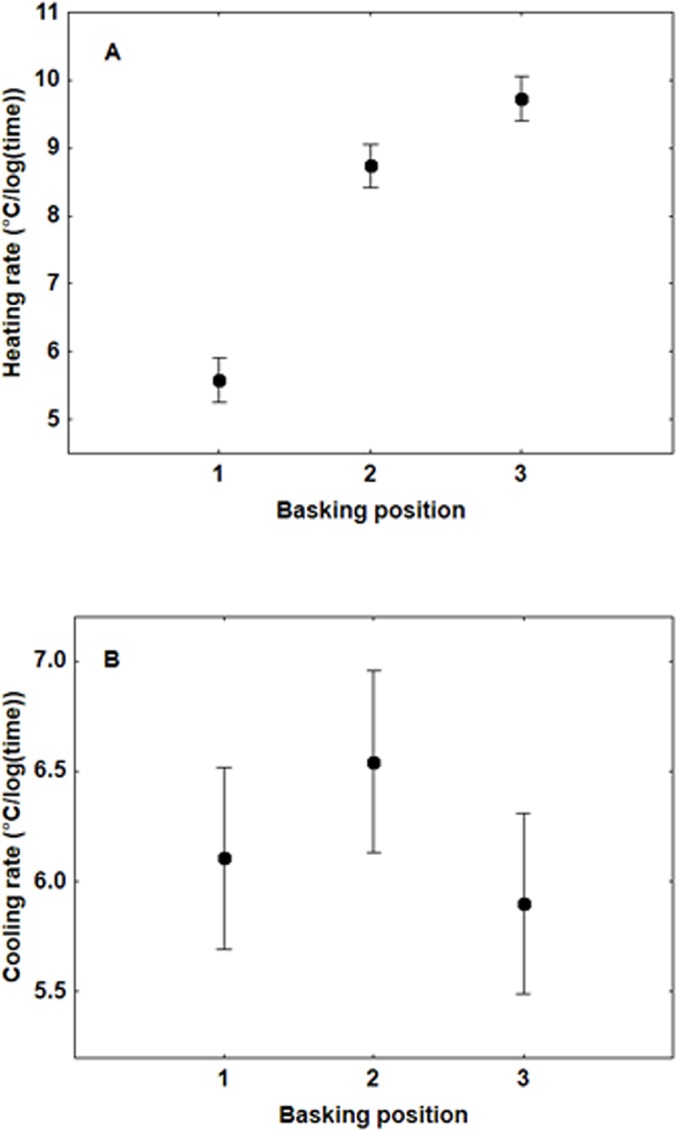
Heating (A) and cooling (B) rates of *P*. *icarus* butterflies in three basking positions (means +/- S.E.) (Heating: F_2,86_ = 414.15, P < 0.0001; Cooling: F_2,86_ = 4.74, P = 0.011). For basking positions see Fig 1.

There were no differences between the sexes in heating rates (*F*_1,42_ = 7.41, *P* = 0.081). Basking position was highly significant (*F*_2,86_ = 414.15, *P*<0.0001) with a higher heating rate the more open the wings (Fig 4A). There were no differences between the sexes in cooling rates (*F*_1,42_ = 4.37, *P* = 0.284). Wing position was significant for cooling rate (*F*_2,86_ = 4.74, *P* = 0.011); post hoc Tukey tests showed that the only significant difference was between BP2 and BP3 (Fig 4B), with faster cooling with half open wings.

In order to investigate the relative contributions of melanisation and weight, or whether other (unmeasured) factors that vary between individuals have greater influence over our response variables, three models are compared (Table 2). Model 1 containing sex (fixed), individual nested within sex (random) and basking position (fixed). Model 2 had sex (fixed), basking position (fixed) and weight as a covariate and Model 3 had sex (fixed), basking position (fixed) and melanisation as a covariate. Melanisation did not affect maximum temperature or heating rate. Weight did not affect heating rate, but did affect the maximum temperature attained (Table 2); heavier individuals, across both sexes, reached higher temperatures. Melanisation had a small positive effect on cooling rates (Effect size: *ƞ^2^* = 0.032; Table 2), but both weight and melanisation explained much less of the variation in cooling rates than individual variation. Model 1 always explained more variation than either model 2 or 3 (Table 2) indicating that other unmeasured factors between individuals explained more variation in our response variables than either weight or melanisation.

**Table 2:** Comparison of the models for maximum temperature, heating rates and cooling rates for both weight and melanisation.

					Whole model				
Dependent variable: Maximum temperature	SS model	df model	SS residual	df residual	F	p	Adj R^2^	Model term of interest and direction of effect	Significance
Model 1: sex + basking position + individual(sex)	2133.08	45	110.53	86	36.88	< 0.0001	0.925	individual(sex)	F_42,86_ = 2.27; p = 0.0007
Model 2: sex + basking position + weight	2024.82	4	218.79	127	293.83	< 0.0001	0.899	weight (+)	F_1,127_ = 8.226; p = 0.0048
Model 3: sex + basking position + melanisation	2010.96	4	232.66	127	274.43	< 0.0001	0.893	melanisation (-)	F_1,127_ = 0.167; p = 0.6830
Summary: weight has a significant positive effect on maximum temperatures reached, but melanisation has not.									
Dependent variable: Heating rate									
Model 1: sex + basking position + individual(sex)	518.68	45	50.32	86	19.70	< 0.0001	0.865	individual(sex)	F_42,86_ = 3.95; p < 0.0001
Model 2: sex + basking position + weight	423.12	4	145.88	127	92.09	< 0.0001	0.736	weight (+)	F_1,127_ = 1.36; p = 0.245
Model 3: sex + basking position + melanisation	423.16	4	145.85	127	92.12	< 0.0001	0.736	melanisation (+)	F_1,127_ = 1.39; p = 0.240
Summary: neither weight nor melanisation have an effect on heating rates									
Dependent variable: Cooling rate									
Model 1: sex + basking position + individual(sex)	169.93	45	85.70	86	3.79	< 0.0001	0.489	individual(sex)	F_42,86_ = 3.73; p < 0.0001
Model 2: sex + basking position + weight	14.53	4	241.1	127	1.91	0.1121	0.027	weight (+)	F_1,127_ = 0.37; p = 0.547
Model 3: sex + basking position + melanisation	21.46	4	234.17	127	2.91	0.0241	0.055	melanisation (+)	F_1,127_ = 4.135; p = 0.044
Summary: melanisation has a positive effect on cooling rates									
Quantitative comparison of models*	Maximum temperature				Heating Rate			Cooling Rate	
Model 1 vs Model 2	F_41,86_ = 2.05; p = 0.0026				F_41,86_ = 3.98; p < 0.0001			F_41,86_ = 3.80; p < 0.0001	
Model 1 vs Model 3	F_41,86_ = 2.32; p = 0.0005				F_41,86_ = 3.98; p < 0.0001			F_41,86_ = 3.63; p < 0.0001	

* Quantitative comparison of the models was done by calculating the F ratio as: F = [(difference in SS explained)/(difference in df)] / (residual mean square of model 1)

## Discussion

Our results indicate that variation in wing melanisation only plays a minor role in heating rates or in the maximum achieved body temperature in the small sized butterfly, *P*. *icarus* under radiation and temperature conditions approximating to those which are marginal for activity in the field. Warming rates are maximised with fully open wings fully exposing the body and weight is important; heavier individuals can attain higher temperatures than lighter individuals. Further evidence for the lack of a significant role for melanisation having no effect on warming rates in this butterfly is provided by males and females having similar warming rates despite males being less melanised than females. Posture also affected cooling rates; those with half open wings cooled the most rapidly. Convective currents around the body may be reduced with closed or fully open wings possibly explaining the slower cooling rates in those two basking positions. Weight had no effect on cooling rate, but melanisation had a small positive effect.

The wings of lycaenid butterflies have two scale types. Cover scales, comprising ridges and cross members, are not melanized and act as photonic crystals reflecting light [31]-[32], [38]. The architecture of these scales determines which wavelengths are reflected. In *P*. *icarus* they primarily reflect short wavelengths (UV/blue). Basal scales are simpler in structure; they do not reflect short wavelength light and melanin within the scales absorbs light [31]. Variation in the proportion of these scale types should influence warming rates, dependent on basking method and wing position, if melanisation influences thermoregulation. However, we found no effect of variability in melanisation (caused by differences in the proportions of scales types) on warming rates in *P*.*icarus*, indicating that variation in wing colour may interact with other, visual, wing functions.

It has been suggested that *P*. *icarus* is a reflectance basker [3], though field evidence is not consistent with this [27]. Our laboratory studies also demonstrate that this is not the case as maximum warming rates were achieved with fully open wings, a basking posture that will not reflect incident radiation onto the body. This is a posture that is consistent with an open-winged absorbance basking posture. There were no differences between the warming rates of the sexes, despite females being darker than males, and no effect of melanisation within the sexes. Thus, the species cannot be classified as an absorbance basker but the higher warming rate for open winged individuals is consistent with posture reducing convection currents around the body. Although the wings of Lycaenidae with a greater proportion of melanised scales have been demonstrated to become warmer than wings with a high proportion of reflective cover scales [39] our results indicate that wing warming rates are of no significance to thoracic temperature. In our experiments there were differences between the maximum temperatures and warming rates of dead and live individuals. Although dead individuals achieved higher final temperatures and had faster warming rates than live individuals the overall pattern of results in relation to size, sex and basking posture is the same for both states. The most likely explanation, consistent with our identifying weight as important to warming, is that dead individuals dehydrated during the course of the experimental runs, effectively lowering their mass in comparison to live individuals.

Our experimental set-up was designed to approximate light intensities in marginal conditions in the field for *P*. *icarus* activity [27]-[28]. Although it could be suggested that we are potentially underestimating the heating effect from the warming effects of the Halogen light sources because of the increased IR output in comparison to sunlight, any differential effect of melanisation would be more pronounced than we have found here. In our experimental conditions the most important factors influencing warming rate were posture and weight. This is consistent with temperature being dependent on exposing the thorax to solar radiation to raise thoracic temperature and using the wings to reduce convective currents around the body. Cooling rates are also dependent on posture, individuals cooling most rapidly with half open wings, a posture which is of low efficiency for minimizing convective cooling in comparison to holding the wings closed or fully open [23].

In the British Isles, *P*. *icarus* differs in body temperature for activity between northern and southern populations [28] but variation in ambient temperature or solar radiation in the field does not influence this body temperature as much as does the microhabitat temperature of basking sites, which are characterised by bare ground with short vegetation in small pockets surrounded by taller vegetation [28]. Such sites tend to be relatively warm. We therefore suggest that for butterflies of the same or of a lower mass than *P*. *icarus*, wing colouration may play no role in thermoregulation. For *P*. *icarus* there is also dependence on warm sheltered locations for raising body temperature. The thermal excesses (body—ambient temperature) obtained by *P*. *icarus* in the field [27]-[28] are also small compared to larger species, corroborating this idea.

Butterfly wing colour (and pattern) has roles in predator avoidance and evasion, intraspecific communication and mate recognition [21] as a result of which constraints may arise. On the basis of our experimental results we suggest that thermal functions of wing colour are minimal for *P*. *icarus* and the main factors involved in wing colour are signal functions involving predators and mate recognition. *Polyommatus icarus* has been described as being very variable in wing colouration (especially of females) and size both geographically [40] and within populations [27]. This may be because wing function is freed from thermoregulatory constraints. Studies of other species where wings have thermoregulatory function have demonstrated that variation in those wing areas of particular thermal function may vary independently of other wing areas [21]. In *P*. *icarus* basal wing melanisation is correlated with melanisation of the central wing area (this study) and with the whole wing area [27], indicating that there may no such uncoupling. Our studies also reveal no thermal effect of variation in melanisation of this butterfly even if there is no uncoupling of wing area functions.

In the British Isles individuals of *P*. *icarus* in the north are larger and brighter than in the south. Although northern populations are univoltine, and with longer development periods are expected to be larger [41], thermoregulatory constraints on the adult stage may also select against small size in northern populations; maximum body temperatures are positively related to weight. There may thus be a minimal size in marginal cool and cloudy conditions to allow prolonged activity. The requirement to be large (thermally stable) in cool locations may therefore act as an additional constraint on generation number and further explain evidence that suggests northern populations have an obligate univoltine life-history strategy [42]. This thermal constraint on size may also explain why the shift from a bivoltine to univoltine strategy is further south in the more oceanic British Isles (cooler and cloudier summer conditions; Maritime Temperate) than in Sweden with a more continental climate (higher summer temperatures and more sunshine hours; Warm Summer Continental) (Köppen-Geiger Climate Classification [43]).

We detected no differences in heating or cooling rates between males and females. In the studied samples the two sexes did not differ in weights, though males had larger wings (and thus slightly lighter bodies). Despite having faster development times than females, and thus a protandrous strategy, males accumulate body mass at faster rate than females and do not emerge lighter than females [42]. As body weight is of importance for thermoregulation in this small butterfly, we suggest that larval feeding strategies are intricately linked (via food accumulation) with the requirements for adult thermoregulation as well as development speed being linked with a protandrous strategy.

The pattern of variation in blueness and brightness in *Polyommatus* butterflies is the result of differences in the proportions of cover and basal scale types [39]. Brightness in northern regions, resulting from an increased proportion of cover scales, can be explained by requirements to maximise apparency in conditions where activity is less frequent than in southern areas [27]. Apparency may come with a cost, vulnerability to predation, but with no thermal interaction. The roles of ecological factors such as variation in population size and visually hunting predators in determining such costs warrant detailed field studies as do the developmental mechanisms and their interactions with conditions during development underlying the production of different scale types in the Lycaenidae.

It has been suggested [39] that increases in the proportion of dark wing scales with altitude and latitude in some Lycaenidae is of thermoregulatory significance. We suggest from our laboratory studies, combined with earlier evidence from studies of warming in the field [27], [29] that in *P*. *icarus* at least, this is not the case. Colour lightness of the dorsal wing surfaces may be related to functions other than thermoregulation in butterflies [44], being an exaptation of a trait evolved in response to past environments. The polyommatine section of the Lycaenidae is species rich, with a widespread distribution and complex distribution histories [26], and morphologically variable, especially in wing colouration. We suggest that this variation might be linked to a lack of a thermoregulatory function of wing colour because of the typical small mass of these species. In addition mate recognition is primarily based on males recognising conspecifics by wing colour. Where closely related *Polyommatus* species co-occur there is evidence for phenological shifts which have been linked to the need to maintain pre-zygotic isolation mechanisms where there are visual mate-recognition systems [11]. We suggest that such phenological shifts may be easier to make when there is a minimal or absent thermoregulatory function associated with wing colouration. Additionally, an absence of thermoregulatory function provides a minimal constraint on maintaining species-specific optical signalling for mate recognition between thermally variable environments. In a wider context the relatively high variation in wing colour and pattern of the polyommatine section may be partly related to minimal or absent thermoregulatory function of wing colouration. If this is the case then there is an expectation that pattern and colour divergence, and thus within taxon diversity, may be greater in low mass butterflies than in those with higher mass in locations where there are thermal constraints on activity.

## Supporting Information

S1 Table Measured temperatures of Polyommatus icarus_ heating and cooling. (XLSX)

## References

[1] Watt WB. Adaptive significance of pigment polymorphisms in *Colias* butterflies. I. Variation of melanin pigment in relation to thermoregulation. Evolution 1968; 22: 437–458. 28564757 10.1111/j.1558-5646.1968.tb03985.x

[2] Silberglied RE. Visual communication and sexual selection among butterflies In: Vane-Wright RI, Ackery PR, editors. The biology of butterflies. Princeton: Princeton University Press; 1984 pp. 207–223.

[3] Kingsolver JG. Butterfly thermoregulation: Organismic mechanisms and population consequences. J Res Lep. 1985; 24: 1–20.

[4] Dennis RLH, Shreeve TG. Butterfly wing morphology variation in the British Isles—The influence of climate, behavioral posture and the hostplant habitat. Biol. J. Linn. Soc. 1989; 38: 323–348.

[5] Vane-Wright RI, Boppré M. Visual and chemical signalling in butterflies: Functional and phylogenetic perspectives. Phil Trans R Soc Lond Ser B Biol Sci. 1993; 340: 197–205.

[6] Brunton CFA, Majerus MEN. Ultraviolet colours in butterflies: intra- or inter-specific communication? Proc R Soc Lond Ser B Biol Sci 1995; 260: 199–204.

[7] Fordyce JA, Nice CC, Forister ML, Shapiro AM. The significance of wing pattern diversity in the Lycaenidae: mate discrimination by two recently diverged species. J Evol Biol. 2002; 15: 871–879.

[8] Lyytinen A, Brakefield PM, Mappes J. Significance of butterfly eyespots as an anti-predator device in ground-based and aerial attacks. Oikos 2003; 100: 373–379.

[9] Rutowski RL, Nahm AC, Macedonia JM. Iridescent hindwing patches in the Pipevine Swallowtail: differences in dorsal and ventral surfaces relate to signal function and context. Funct Ecol.2010; 24: 767–775

[10] Kemp DJ, Rutowski RL. The role of coloration in mate choice and sexual interactions in butterflies. Adv Stud Behav 2011; 43: 55–92.

[11] Bálint Z, Kertész K, Piszter G, Vértesy Z, Biró LP. 2012. The well-tuned blues: the role of structural colours as optical signals in the species recognition of a local butterfly fauna (Lepidoptera: Lycaenidae: Polyommatinae). J R Soc Interface 2012; 9: 1745–1746. 10.1098/rsif.2011.0854 22319114 PMC3385757

[12] Shreeve TG, Dennis RLH. The development of butterfly settling posture—the role of predators, climate, hostplant-habitat and phylogeny. Biol J Linn Soc. 1992; 45: 57–69.

[13] Rutowski RL. Evidence for mate choice in a sulphur butterfly (*Colias eurytheme*). Z Tierpsychol. 1985; 70: 103–114.

[14] Kemp DJ. Female butterflies prefer males bearing bright iridescent ornamentation. Proc R Soc Lond Ser B Biol Sci. 2007; 274: 1043–1047. 10.1098/rspb.2006.0043 PMC2124467 17284412

[15] Lyytinen A, Brakefield PM, Lindström L, Mappes J. Does predation maintain eyespot plasticity in *Bicyclus anynana*? Proc R Soc Lond Ser B: Biol Sci. 2004; 271: 279–283. 10.1098/rspb.2003.2571 PMC1691594 15058439

[16] Morehouse NI, Rutowski RL. In the eyes of the beholders: female choice and avian predation risk associated with an exaggerated male butterfly color. Am Nat. 2010; 176: 768–784. 10.1086/657043 20942644

[17] Olofsson M, Vallin A, Jakobsson S, Wiklund C. Marginal eyespots on butterfly wings deflect bird attacks under low light intensities with UV wavelengths. PLoS ONE 2010; 5: e10798 10.1371/journal.pone.0010798 20520736 PMC2875403

[18] Kingsolver JG. Experimental manipulation of wing pigment pattern and survival in western white butterflies. Am Nat. 1996; 147: 296–306.

[19] Van Dyck H, Matthysen E, Dhondt AA. The effect of wing colour on male behavioural strategies in the speckled wood butterfly. Anim Behav. 1997; 53: 39–51.

[20] Trullas SC, van Wyck JH, Spotila JR. Thermal melanism in ectotherms. J Therm Biol. 2007; 32: 235–245.

[21] Shreeve TG, Konvicka M, Van Dyck H. Functional significance of butterfly wing morphology variation In: Settele J, Shreeve T, Konicka M, Van Dyck H, editors. Ecology of Butterflies in Europe. Cambridge: Cambridge University Press; 2009 pp. 171–188.

[22] Oliver JC, Robetson KA, Monteiro A. Accommodating natural and sexual selection in butterfly wing pattern evolution. Proc R Soc Lond Ser B: Biol Sci. 2009; 276: 2369–2375. 10.1098/rspb.2009.0182 PMC2690465 19364741

[23] Kingsolver JG, Moffat RJ. Thermoregulation and the determinants of heat transfer in *Colias* butterflies. Oecologia 1982; 53: 27–33. 28310599 10.1007/BF00377132

[24] Heinrich B. Comparative thermoregulation of four montane butterflies of different mass. Physiol Zool.1986; 59: 616–626.

[25] Eliot JN. The higher classification of the Lycaenidae (Lepidoptera): a tentative arrangement. Bull Br Mus Nat Hist (Ent). 1973; 28: 371–505.

[26] Vila R, Bell CD, Macniven R, Goldman-Huertas B, Ree RH, Marshall CR, et al 2011. Phylogeny and palaeoecology of *Polyommatus* blue butterflies show Beringia was a climate-regulated gateway to the New World. Proc R Soc Lond Ser B: Biol Sci. 2011; 278: 2737–2744 10.1098/rspb.2010.2213 PMC3145179 21270033

[27] Howe PD. The ecological consequences of morphological variation in the common blue butterfly *Polyommatus icarus* (Rott.) in the United Kingdom. Ph.D. Thesis. Oxford Brookes University. 2004. Available: http://ethos.bl.uk/Home.do

[28] Howe PD, Bryant SR, Shreeve TG. Predicting body temperature and activity of adult *Polyommatus icarus* using neural network models under current and projected climate scenarios. Oecologia 2007; 153: 857–869. 17587061 10.1007/s00442-007-0782-3

[29] Rasband WS. Image J, 1997–2010. U.S. National Institutes of Health, Bethesda, Maryland, USA. Available: http://imagej.nih.gov/ij/

[30] Ghiradella H. Hairs, bristles and scales In: Locke M. editor. Microscopic anatomy of invertebrates. Wilmington: Wiley–Liss Publishers; 1998 pp. 257–287.

[31] Bálint Z, Vértesy Z, Kertész Z, Biró LP. Scanning electron microscopic investigations in butterfly wings: detecting scale micro- and nanomorphology and understanding their functions Curr Issues Multidisc Microsc Res Educ. 2004: 87–92.

[32] Kertész K, Bálint Z, Vértesy Z, Márk GI, Lousse V, Vigneron J-P. Photonic crystal type structures of biological origin: Structural and spectral characterization. Curr App Phys. 2006; 6: 252–258.

[33] Talloen W, Van Dyck H, Lens L. The cost of melanisation: butterfly wing coloration under environmental stress. Evolution 2004; 58: 360–366. 15068352 10.1111/j.0014-3820.2004.tb01651.x

[34] Wasserthal LT. The role of butterfly wings in regulation of body temperature. J Insect Physiol. 1975; 21: 1921–1930.

[35] Klingenberg C. Analysis of organismal form: An introduction to morphometrics.2008. Available: http://www.flywings.org.uk/MorphoCourse/index.htm

[36] Van Dyck H, Matthysen E. Thermoregulatory differences between phenotypes in the speckled wood butterfly: hot perchers and cold patrollers? Oecologia 1998;114: 326–334. 28307775 10.1007/s004420050454

[37] R Development Core Team. 2013. R: a language and environment for statistical computing R Foundation for Statistical Computing, Vienna 2013 Available: http://www.R-Project.org

[38] Ingram AL, Parker AR. A review of the diversity and evolution of photonic structures in butterflies, incorporating the work of John Huxley (The Natural History Museum, London from 1961 to 1990). Phil Trans R Soc Lond Ser B: Biol Sci. 2008; 363: 2465–2480. 10.1098/rstb.2007.2258 18331987 PMC2606806

[39] Biró LP, Bálint Z, Kertész K, Vértesy Z, Márk GI, Z. E. Horváth ZE, et al Role of photonic crystal-type structures in the thermal regulation of a lycaenid butterfly sister species pair. Phys Rev E. 2003; 67:71–77. 10.1103/PhysRevE.67.021907 12636715

[40] Emmet AM, Heath J. 1982 Polyommatus icarus In:The Moths and Butterflies of Great Britain and Ireland. Volume 7 (I) Hesperiidae—Nymphalidae pp. 249–252. Harley Books: Colchester.

[41] Nygren GH, Bergström A, Nylin S. Latitudinal body size clines in the butterfly *Polyommatus icarus* are shaped by gene-environment interactions. J Insect Sci. 2008; 8: 10.1673/031.008.4701

[42] De Keyser R. 2012. Spatial structuring in trait variation in *Polyommatus icarus* in a functional context. Ph.D. Thesis. Oxford Brookes University. 2012. Available: http://ethos.bl.uk/OrderDetails.do?did=1&uin=uk.bl.ethos.579548.

[43] Peel MC, Finlayson BL, Mcmahon TA. Updated world map of the Köppen–Geiger climate classification. Hydrol Earth Syst Sci. 2007; 11: 1633–1644.

[44] Zeuss D, Brandl R, Brändle M, Rahbek C, Brunzel S. Global warming favours light coloured insects in Europe. Nature Comms. 2014; 5: Art.3874 10.1038/ncomms4874 PMC4050276 24866819

